# The Scheme to Level All Distinctions in Medicine, Voiced by Senator Looney

**Published:** 1907-04

**Authors:** 


### EDITORIAL DEPARTMENT.

### THE SCHEME TO LEVEL ALL DISTINCTIONS IN MEDICINE, VOICED BY SENATOR LOONEY. MINORITY RULE. THE TAIL WAGS THE DOG.

It seems to me we are face to face with a proposition that strikes at the fundamental principle of professional ethics, and which must be abhorrent to every physician educated in the old school thought, and who venerates the high aims, ideals and traditions of scientific medicine.

When the Mixed Board Bill—published herewith, with all the amendments which were put in by the House on its recommittal—was on its final passage in the Senate, Senator Alexander said: "It is not right that four or five physicians of Texas should defeat the desires of thousands of Texans who patronize the other schools, and work an injustice against the minority schools."

He might with equal propriety have said: "It is not right that four or five Texas physicians should defeat the desires and disregard the voice and authority of the State Medical Association so emphatically expressed by its House of Delegates at its Fort Worth meeting last April." The proposition to create a mixed board on which shall be represented all of the so-called "schools" was most emphatically condemned; and, instead, a bill requiring the Governor to appoint a board of eleven physicians (without suggestion or dictation from any source, or reference to any "school") was unanimously adopted, and the Legislative Committee (President and Secretary *ex officio* members were instructed to present it to the Legislature. They did so, and it passed the Senate with many objectionable amendments, to all of which the committee agreed. It went to the House, when, lo! this committee and its advisers bargained with the representatives of the osteopaths, the homeopaths, the physio-medicals, the eclectics, and God knows whom else, for representation on said board.

In so doing they nullified the action of the House of Delegates from whom they derive their authority, and restored, practically, the features of the bill which were so emphatically repudiated and defeated by the House of Delegates.

Has this committee of five supreme power? Have they authority to disregard the action of the House and the will and the voice of the vast majority of the members? Have they the right to commit nine-tenths of the Association to an objectionable policy of homologation, and a bill recognizing all the irregulars?

Under the organization of the State Association the House is composed of one representative of each county society, and the House is supposed to represent the wishes of its constituents. That it does not always do so, I have reason to know. The "Scientific branch"—composed of about nine-tenths of the members—has no voice in any matter except as expressed through the House. And in this instance the voice has been disregarded—and the House and the Scientific branch,—the whole Association,—have been committed to a policy which I believe that nine-tenths of the members would repudiate if given an opportunity to express themselves. Verily, it is a high-handed and, I think, an unwarranted exercise of authority which should be repudiated at the meeting next month.

One feature (happily knocked out at the Governor's request, as Mr. Looney says), was that providing for a list of ten from each "school" having a State organization, from which lists the Governor was to make his selection. What chance had a member of the Association not in accord with the policy of the homologation, to get on that list? None whatever; the bosses would see to that. (Now, do not misunderstand me. The writer having long since retired from practice, is not eligible, and under no circumstances, if eligible, would he serve on such a Board.) He has been consistent, at least, and recalls the time when Dr. Ross of Brenham was tried for heresy because he served with a homeopath on a local board. He recalls also the action of the State Association in declaring they would expel any member for consulting with a homeopath.

But there are those in the House who would accept such appointment, but who, because they do not fall in with what radical members call the "new dispensation," would not be recommended. It is a kind of gag. Put none but "new dispensation" men in the House is the slogan of reform; men who will carry out the behests of the Dictator Simmons, whose scheme of unifying involves the breaking down of all barriers between regular medicine and the fungus growths represented by new and so-called "schools"—a homologating with the irregulars, who, as I have often pointed out, are, from the standpoint of medical ethics, quacks. The dictator of the Seventh District, dictated, nominated and caused to be elected, a delegate of his selection. In so doing he violated the by-laws, which state that there shall be no nominations.

That what I state above is true, that the State Association is committed by its Legislative Committee and a few advisers, to a policy which I really believe is repugnant to a large majority of members, and that, too, in utter disregard of the will of that majority as expressed through its House of Delegates, I quote from Senator Looney's speech in the Senate on the final passage of the amended Act. Of course, it is *ex cathedra*. Mr. Looney must been given to understand that it was the policy and desire of the State Medical Association to break down all barriers between the regular medical profession and the numerous "pathies." Emphatically, it is not; but it is the policy of a clique, dominated by the Octopus.

Senator Looney said * * * "the intention is to get a medical bill, but every man can not get everything he wants, and the Governor had given notice that he would veto the bill unless it was changed, and that it had been changed to meet every expressed idea of the Governor so as to have such a bill. The Senator then justified his bill as *uniting for the first time the medical schools, wiping out the lines of demarkation, uniting the brethren and moulding them into a consolidated board!*"—*Statesman.*

Dost thou like the picture?

Will the physicians of Texas stand for this? Shades of Davis and Hamilton go weep!

That this is a part of the scheme dictated from Chicago to create a Medical Journal Trust, I verily believe; and the State Medical Association has been *played into the hands of the Octopus* by a very small minority. The tail wags the dog.

Will the large element of ethical and self-respecting men of the Association,—those who venerate the traditions, achievements and high ideals of legitimate, scientific medicine, and who, like the writer, know but one "school" of medicine,—and who always uphold the standard and demand a conformity to its principles, consent to be so handed over to humiliation and degradation by a handful of over-zealous but unthinking younger men? Delivered without protest, like a herd of purchased cattle, and, perforce, just as mute? God forbid!

Shall this unwise, unpopular state of affairs be continued? Or shall we have once a year, as formerly, and for thirty-five years, a medical convention where every member can have a voice, and register his vote for or against matters that so deeply touch the honor, welfare,—nay, perpetuity of the regular medical profession? Shall Simmons' lieutenants—the subordinate Bosses called "Councilors,"—strikes, to do the behests of the Octopus and its Walking Delegate, McCormack, pack the House of Delegates with instructed and pliant delegates? Will the physicians of Texas be longer dictated to as to what they shall read, and what they shall do, and what they shall prescribe? Or will they assert their God-given rights to think and to act for themselves, and to be heard in the councils of a State association?

